# Climate change impacts on the predicted geographic distribution of *Betula tianschanica* Rupr

**DOI:** 10.3389/fpls.2025.1528255

**Published:** 2025-03-11

**Authors:** Hang Zhou, Ao Li, Xuequn Luo, Jiafeng Wang, Yihong Xie, Zhongping Lin, Donglai Hua

**Affiliations:** ^1^ School of Resources and Environmental Engineering, Mianyang Teachers’ College, Mianyang, China; ^2^ School of Life Science and Technology, Minshan Biodiversity Monitoring and Analysis Laboratory of Giant Panda National Park, Mianyang Teachers’ College, Mianyang, China; ^3^ College of Life Sciences, Peking University, Beijing, China

**Keywords:** *Betula tianschanica* Rupr., MaxEnt, RF, environmental factors, predicted geographic distribution

## Abstract

**Introduction:**

*Betula tianschanica* Rupr. is distributed in regions such as China, Kyrgyzstan, and Tajikistan. Owing to the impacts of climate change, it is increasingly threatened by habitat fragmentation, resulting in a precipitous decline in its population. Currently listed as endangered on the Red List of Trees of Central Asia, this species is predominantly found in the Tianshan Mountains. Examining the influence of climate change on the geographical distribution pattern of *Betula tianschanica* is crucial for the management and conservation of its wild resources.

**Methods:**

This study employed two models, maximum entropy (MaxEnt) and random forest (RF), combined with 116 distribution points of *Betula tianschanica* and 27 environmental factor variables, to investigate the environmental determinants of the distribution of *Betula tianschanica* and project its potential geographical distribution areas.

**Results:**

The MaxEnt model and the RF model determined the primary environmental factors influencing the potential distribution of *Betula tianschanica*. The MaxEnt model showed that the percentage of gravel volume in the lower soil layer and elevation are the most significant, while the RF model considered elevation and precipitation of the wettest quarter to be the most crucial. Both models unanimously asserted that elevation is the pivotal environmental element affecting the distribution of *Betula tianschanica*.

The mean area under the curve (AUC) scores for the MaxEnt model and RF were 0.970 and 0.873, respectively, revealing that the MaxEnt model outperformed the RF model in predictive accuracy. Consequently, the present study employed the estimated geographical area for *Betula tianschanica* modeled by the MaxEnt model as a reference. Following the MaxEnt model’s projected outcomes, *Betula tianschanica* is mainly located in territories such as the Tianshan Mountains, Ili River Basin, Lake Issyk-Kul, Turpan Basin, Irtysh River, Ulungur River, Bogda Mountains, Kazakh Hills, Lake Balkhash, Amu River, and the middle reaches of the Syr River.

Within the MaxEnt model, the total suitable habitat area exhibits growth across all scenarios, with the exception of a decline observed during the 2041–2060 period under the SSP2-4.5 scenario. Remarkably, under the SSP58.5 scenario for the same timeframe, this area expands significantly by 42.7%. In contrast, the RF model demonstrated relatively minor fluctuations in the total suitable habitat area, with the highest recorded increase being 12.81%. This paper recommends establishing protected areas in the Tianshan Mountains, conducting long-term monitoring of its population dynamics, and enhancing international cooperation. In response to future climate change, climate refuges should be established and adaptive management implemented to ensure the survival and reproduction of *Betula tianschanica*.

## 1 Introduction

Climate change significantly impacts plant growth, development, physiology, and ecosystems, altering their geographical distribution (He et al., 2021; Hu X-G. et al., 2024). The Sixth Coupled Model Intercomparison Project (CMIP6) forecasts a temperature increase of 2%–6°C across Central Asian regions by the close of the 21st century (Zhou et al., 2019). This warming could modify plant habitats, leading to reduced populations and even endangerment for some species, thereby accelerating the global loss of biodiversity (Leadley et al., 2010). Central Asia’s complex geography, including the largest non-zonal arid region, makes it highly sensitive to climate change (Cowan, 2007). The region’s plant distribution is shifting from higher to lower elevations due to temperature and precipitation changes (Propastin et al., 2008; Frank et al., 2015; Kong et al., 2017; Lamchin et al., 2018). Beyond climatic factors, the attributes of soil, topography, and elevation are equally pivotal in shaping plant distribution (Elsen et al., 2020; Gałązka et al., 2022; Hu Z. et al., 2024; Tenaw et al., 2024). Notably, changes in elevation exert significant influences by modulating temperature, humidity, and soil nutrient profiles, thus emerging as a core determinant in defining the spatial distribution of plant species (Simin et al., 2022; Tian et al., 2023; Ye et al., 2024). Amid the backdrop of worldwide climate change, the survival of Central Asian plants is under severe threat. Exploring how climate change influences ecological distribution is critical for the region’s conservation strategies.

Species distribution models (SDMs) are a method founded on the occurrence records of species and related ecological factors (temperature, humidity, and elevation) to simulate species distribution and ecological needs (Rathore and Sharma, 2023). From the earliest Bioclimatic Analysis and Prediction System (BIOCLIM) (Busby, 1991), it has developed later into the Genetic Algorithm for Rule-Set Production (GARP) (Stockwell, 1999), random forest (RF) model (Chambers et al., 2013), maximum entropy (MaxEnt) model (Austin, 2007), and others. The MaxEnt model is grounded in the theory of maximum entropy. It establishes the connection between species distributions and environmental factors through limited species distribution and environmental data and predicts the distribution probability in unknown areas. Since its introduction by Phillips in 2006 for forecasting species’ potential distributions, the MaxEnt model has emerged as a pivotal tool in ecological research (Phillips et al., 2006). Notably, it excels in scenarios characterized by limited sample sizes and is highly efficient in identifying suitable habitats for endangered species (Cámara-Leret et al., 2019; Koch Liston et al., 2024; Xue et al., 2024). The RF model, proposed by Leo Breiman in 2001, constitutes a type of ensemble learning technique. It builds decision trees using random samples and random feature selection and combines the prediction results of multiple trees to effectively handle the complex non-linear relationships in the data and extract key information. It is particularly suitable for identifying the key environmental factors affecting species distribution and has performed well in simulating the distribution of species such as Arctic whales (Chambault et al., 2022), *Chromolaena odorata* (Adhikari et al., 2023), and *Zelkova carpinifolia* (Koç et al., 2024). Given the limited data points of species distribution in this study and the significant non-linear relationship between environmental factors, the MaxEnt model and the RF model were selected for joint research, aiming to leverage the strengths of both models to enhance the precision and reliability of species distribution forecasting.

*Betula tianschanica* Rupr., a type of tree classified within the genus *Betula*, is part of the Betulaceae family (Zhang et al., 2015) and constitutes a unique pioneer species in Xinjiang, China. Affected by the dry and semi-dry continental climates of the Central Asian region, it primarily thrives in the area of the Tianshan Mountains at an elevation of 1,300–2,500 m. Nowadays, as a result of climate alterations, *B. tianschanica* is facing the threat of habitat fragmentation (Ding et al., 2021). As a consequence, the species remains unclassified in the International Union for Conservation of Nature (IUCN) Red List of Threatened Species owing to inadequate data (Roy et al., 2018). In 2009, *B. tianschanica* was classified as an endangered species in the Red List of Trees of Central Asia (Eastwood et al., 2009). By 2018, it had also been included in the List of Key Protected Wild Plants in the Xinjiang Uygur Autonomous Region and designated as a first-class locally protected plant (China Wild Plant Conservation Association, 2024). *B. tianschanica* is prized not only for its significant ecological value in landscape design, owing to its exceptional cold hardiness and elegant pyramidal form, but also for its multi-seasonal aesthetic appeal. The tree’s decorative peeling white bark and superior summer foliage contribute to its ornamental significance throughout the year (West et al. 2022). Currently, the majority of scholarly work involving *B. tianschanica* centers around dendroclimatology (Zhang et al., 2015), physiological ecology (Li et al., 2001a, b), and phylogenetics (Yang et al., 2023). The principal aims of this study were to 1) determine the primary environmental factors that shape the geographic distribution of *B. tianschanica*, 2) simulate both the contemporary and projected suitable habitats of *B. tianschanica* under varying climate scenarios using dual modeling techniques, and 3) assess the changes in suitable habitat areas and the dynamic distribution characteristics of *B. tianschanica*, thereby offering a robust scientific foundation for the conservation and sustainable management of its wild resources.

## 2 Materials and methods

### 2.1 General situation of study area

Located in the heart of the Eurasian continent, Central Asia comprises the sovereign nations of Kazakhstan, Kyrgyzstan, Tajikistan, Turkmenistan, and Uzbekistan, as well as China’s Xinjiang Uygur Autonomous Region, a provincial-level administrative division. The total area of this expansive region is approximately 560 × 104 km^2^. The terrain in this region is complex, with the high-elevation Altai Mountains, Tianshan Mountains, Pamir Plateau, and Hindu Kush Mountains in the southeast; the Turan Plain and the Caspian Sea littoral in the west; and the Kazakh Hills in the north. The topography is elevated in the southeast and lower in the northwest. The climate is mainly temperate with dry and semi-dry conditions, and certain regions experience influences from Mediterranean and plateau climates. The temperature changes drastically, with a range of 20°C–30°C between day and night. The mean temperature during summer falls between 20°C and 30°C, while in winter, the average temperature drops to below 0°C (Hua et al., 2022). Rainfall across Central Asia is mainly concentrated during the cooler months of winter and spring, varying from more than 1,000 mm in the western Hisar Mountains and Fergana Mountains to less than 100 mm across the eastern regions (Huang et al., 2024). Central Asia is rich and unique in plant resources. Currently, 9,520 species of higher plants have been identified; among these, 20% are unique to the region, classified under 138 families and 1,176 genera. The botanical geography of Central Asia is categorized into five large regions and 33 small regions, with over 65% of the species exhibiting a Central Asian-specific distribution (Zhang et al., 2020).

### 2.2 Distribution point data of *B. tianschanica*

**Figure 1:**
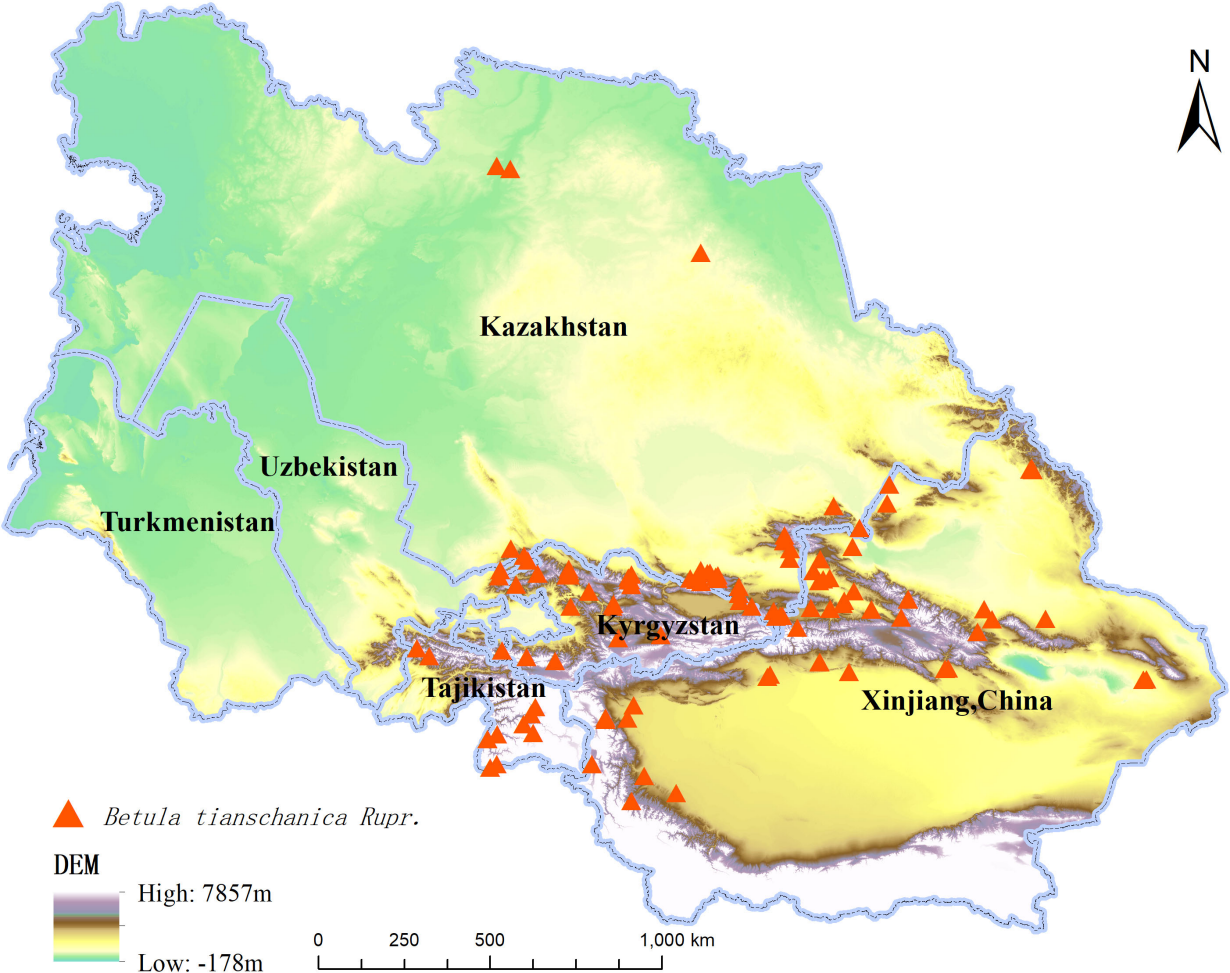
Distribution Records of *Betula tianschanica* Rupr. in Central Asia.

In the current investigation, location data for 197 species of *B. tianschanica* were obtained via the Global Biodiversity Information Facility (GBIF, 2024) and Chinese Virtual Herbarium (2024), with 78 obtained through field investigation. The data were saved in CSV format and imported into the ArcGIS 10.2 software to remove duplicates and invalid entries. To minimize spatial autocorrelation between geographic location coordinates and to maintain consistency with the spatial extent of environmental variables, only one of the two samples existing within the same grid cell was retained. Finally, 116 species’ distribution records (**Figure 1**) were sorted out and saved in ASCII format, which will be used for subsequent distribution model analysis. The national border data were from the Ministry of Natural Resources-Standard Map Service (2024) [Drawing No. GS (2021) No. 5443].

### 2.3 Sources and screening of environmental data

In this study, 19 (Bio1–Bio19) climate data of SSP-RCP2.6 (SSP1-2.6), SSP-RCP4.5 (SSP2-4.5), and SSP-RCP8.5 (SSP5-8.5) were downloaded from the World Climate Data Network in the present period (1970–2000) and the future period (2041–2060 and 2061–2080). The SSP1-2.6 trajectory represents an environmentally sustainable route, with greenhouse gas emissions minimized, and the global temperature is projected to increase by 1.5°C by 2100; SSP2-4.5 is a moderate development trajectory, where emissions of greenhouse gases are at a medium level, and the global temperature is expected to climb by 2.1°C by 2100; SSP5-8.5 is a fossil fuel-intensive path, with significantly increased greenhouse gas output, and the global temperature will see an ascent to 4.7°C by 2100 (Zhou et al., 2021). At the same time, digital elevation model (DEM) data were sourced from the General Bathymetric Chart of the Oceans (GEBCO Compilation Group, 2024) to consider the influence of topography on species distribution. Although many research efforts typically focus on bioclimatic variables and topographic parameters for constructing the model, given that soil characteristics play a crucial role in influencing species distribution, this study also downloaded soil data from the Food and Agriculture Organization’s databases (Fischer et al., 2008) to ensure the comprehensiveness and accuracy of the model. Multicollinearity may lead to inaccuracy and over-fitting of model prediction results. Multicollinearity can result in erroneous model predictions and overfitting. To mitigate this, a Jackknife analysis was conducted for the 36 ecological variables within the MaxEnt model to assess their contribution to model predictions. Variables with no contribution were eliminated, and those with significant contributions were selected. Additionally, ENMtools were utilized for a correlation analysis of soil and climate variables. As a result, 27 environmental variables were chosen for model analysis (**Table 1**), reducing multicollinearity while enhancing the model’s predictive accuracy.

**Table 1:** Environmental variables and codes.

Category	Factor code	Description
Bioclimatic variables	Bio1	Annual mean temperature
	Bio2	Mean diurnal range [mean of monthly (max temp − min temp)]
	Bio4	Temperature seasonality (standard deviation × 100)
	Bio7	Temperature annual range (Bio5–Bio6)
	Bio9	Mean temperature of driest quarter
	Bio10	Mean temperature of warmest quarter
	Bio11	Mean temperature of coldest quarter
	Bio12	Annual precipitation
	Bio14	Precipitation of driest month
	Bio15	Precipitation seasonality (coefficient of variation)
	Bio16	Precipitation of wettest quarter
	Bio17	Precipitation of driest quarter
	Bio18	Precipitation of warmest quarter
	Bio19	Precipitation of coldest quarter
Soil variables	T_GRAVEL^1^	Volume percentage of gravel
	T_ESP	Exchangeable sodium salt
	T_SILT	Silt content
	T_BS	Basic saturation
	S_CEC_CLAY^2^	Cation exchange capacity of cohesive soil
	S_CEC_SOIL	Cation exchange capacity of soil
	S_GRAVEL	Volume percentage of gravel
	S_CASO4	Sulfate content
	S_OC	Organic carbon content
	AWC_CLASS	Soil available water content
Topographical factor	Elevation	Elevation
	Slope	Slope
	Aspect	Aspect

^1^Attribute fields beginning with T_ represent the properties of the upper soil layer (0–30 cm).

^2^Attribute fields beginning with S_ represent the properties of the lower soil layer (30–100 cm).

### 2.4 Construction of the MaxEnt model

The gathered occurrence data and ecological variable information on *B. tianschanica* were converted to ASCII format using ArcGIS 10.2, enabling their integration with MaxEnt 3.4.1 for model processing. In the process, the training subset was allocated 75%, while the testing and validation subset was assigned a 25% share. The number of iterations was fixed at 1,000, and data simulation involved 10 repetitions of the training process (Wu et al., 2022). To assess the limiting influence of individual environmental factors on the spatial distribution patterns of *B. tianschanica*, the Jackknife module in the MaxEnt 3.4.1 software was employed for calculating the relative contribution and the significance through permutation for each ecological parameter. The receiver operating characteristic (ROC) curve was applied to assess the precision of the model’s predictions (Zhu et al., 2017). The area under the curve (AUC) score ranged from 0 to 1.0, and the higher the AUC figure, the better the predictive performance of the model (Wang et al., 2007). The simulation results were output in the Cloglog format and saved in ASCII format, and the remaining parameters used the default settings of the MaxEnt 3.4.1 software.

### 2.5 Construction of the RF model

The random forest model was constructed through the Python software, with *B. tianschanica* as the target tree species. First, the distribution point data of *B. tianschanica* were read from the Shapefile, and the background points without the distribution of the target tree species were generated. Then, the TIFF files of all environmental indicators were read from the specified directory, the environmental indicator data of the species points and background points were extracted, and the outliers were removed. After that, the random forest model was trained using the training set data, the importance of the features was calculated, and the top 10 features were selected based on the importance of retraining the model. Finally, the model was applied to forecast the validation set, the performance metrics were computed, and the ROC graph was created. In addition, the full prediction scope was estimated, and the forecasted data were exported as a new TIFF image.

### 2.6 Comparative analysis of model accuracy

The AUC values were obtained after simulation using the MaxEnt 3.4.1 software and repeated 10 times (**Table 2**). The findings suggest that the average training set AUC for the potential distribution model of *B. tianschanica* under current climate conditions was 0.970, and the average training set AUC for the future climate scenario predictions was above 0.97, demonstrating that the outcomes from the MaxEnt model exhibit high accuracy and reliability and that it is capable of forecasting the potential geographic distribution for *B. tianschanica*. The mean AUC score for the RF model with respect to the prediction results of *B. tianschanica* was 0.873 (**Table 2**), reflecting a considerable predictive precision. The forecasting performance of the RF model was inferior to that of the MaxEnt model, and the results concerning the potential suitable habitat area of *B. tianschanica* were slightly less accurate compared to those of the MaxEnt model.

**Table 2:** AUC scores for predicting the potential distribution of *Betula tianschanica* across various scenarios in different periods by different models.

Model	Period	Scenarios	AUC_mean_
MaxEnt	Current	━━	0.970
	2041–2060	SSP1-2.6	0.973
		SSP2-4.5	0.975
		SSP5-8.5	0.975
	2061–2080	SSP1-2.6	0.975
		SSP2-4.5	0.977
		SSP5-8.5	0.975
RF	Current	━━	0.877
	2041–2060	SSP1-2.6	0.876
		SSP2-4.5	0.876
		SSP5-8.5	0.864
	2061–2080	SSP1-2.6	0.879
		SSP2-4.5	0.877
		SSP5-8.5	0.866

AUC, area under the curve.

## 3 Results and analysis

### 3.1 Key environmental variables influencing species distribution

**Figure 2:**
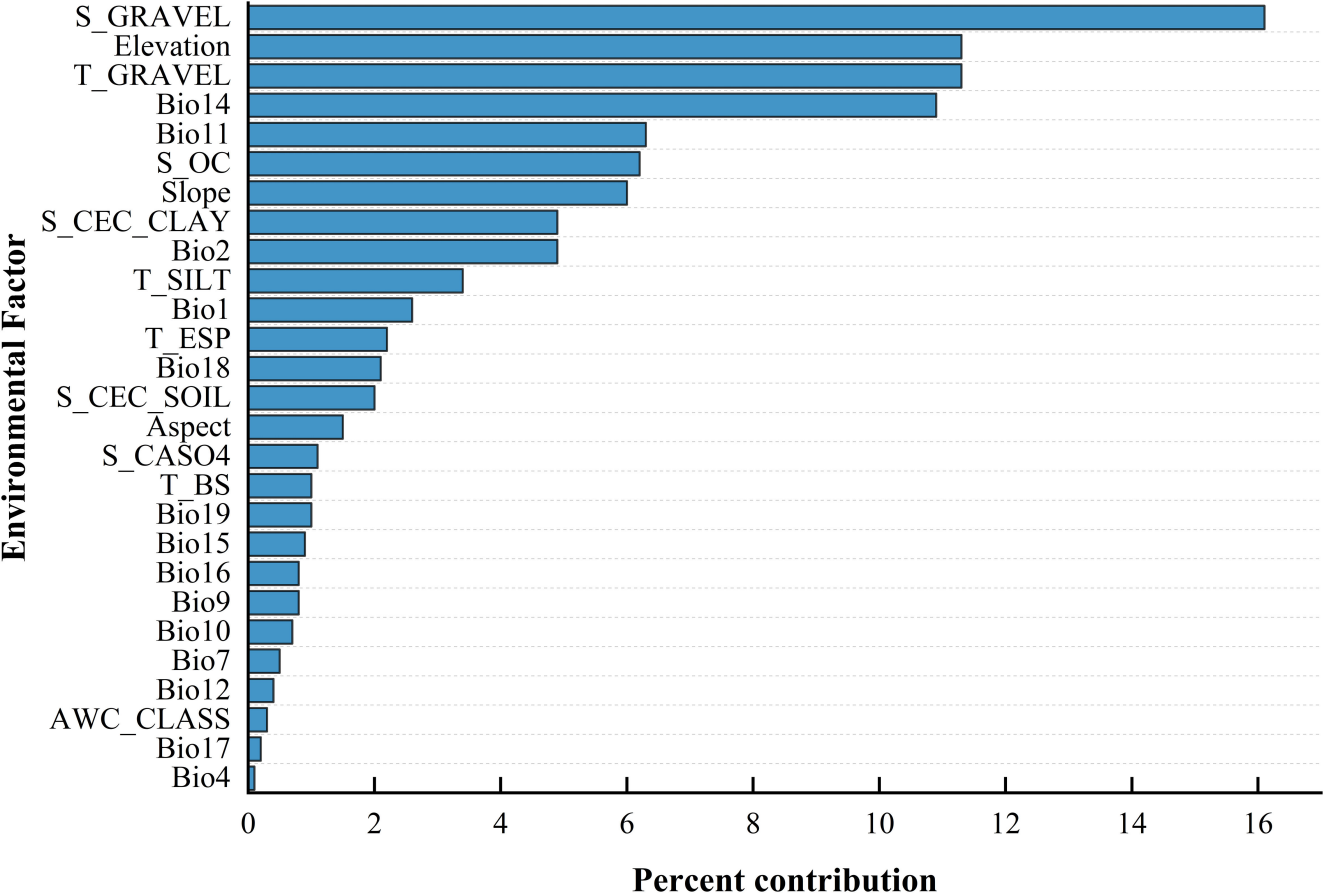
Ranking of Environmental Factor Contributions.

Based on the outcomes of the MaxEnt analysis (**Table 3**) (**Figure 2**), by selecting the six environmental factors with larger contribution rates, it is found that the percentage of gravel volume in the lower layer, the percentage of gravel volume in the upper layer, elevation, precipitation of the driest month, mean temperature of the coldest quarter, and organic carbon content significantly influence the potential distribution of *B. tianschanica*, and their cumulative contribution rate reached 62.1%. The collective contribution of soil attributes (percentage of gravel volume in the lower layer of soil, percentage of gravel volume in the upper layer of soil, and the level of organic carbon within the lower layer of soil) amounted to 33.6%, whereas the aggregate contribution of climatic factors (precipitation of the driest month and mean temperature of the coldest quarter) was 17.2%, and the combined effect of topographical variables (elevation) was 11.3%.

**Table 3:** Percentage of influence from contemporary environmental variables on the MaxEnt model.

Factor code	Percent contribution/%	Factor code	Percent contribution/%
Bio1	2.6	T_GRAVEL	11.3
Bio2	4.9	T_ESP	2.2
Bio4	0.1	T_SILT	3.4
Bio7	0.5	T_BS	1
Bio9	0.8	S_CEC_CLAY	4.9
Bio10	0.7	S_CEC_SOIL	2
Bio11	6.3	S_GRAVEL	16.1
Bio12	0.4	S_CASO4	1.1
Bio14	10.9	S_OC	6.2
Bio15	0.9	AWC_CLASS	0.3
Bio16	0.8	Elevation	11.3
Bio17	0.2	Slope	6
Bio18	2.1	Aspect	1.5
Bio19	1.0		

**Figure 3:**
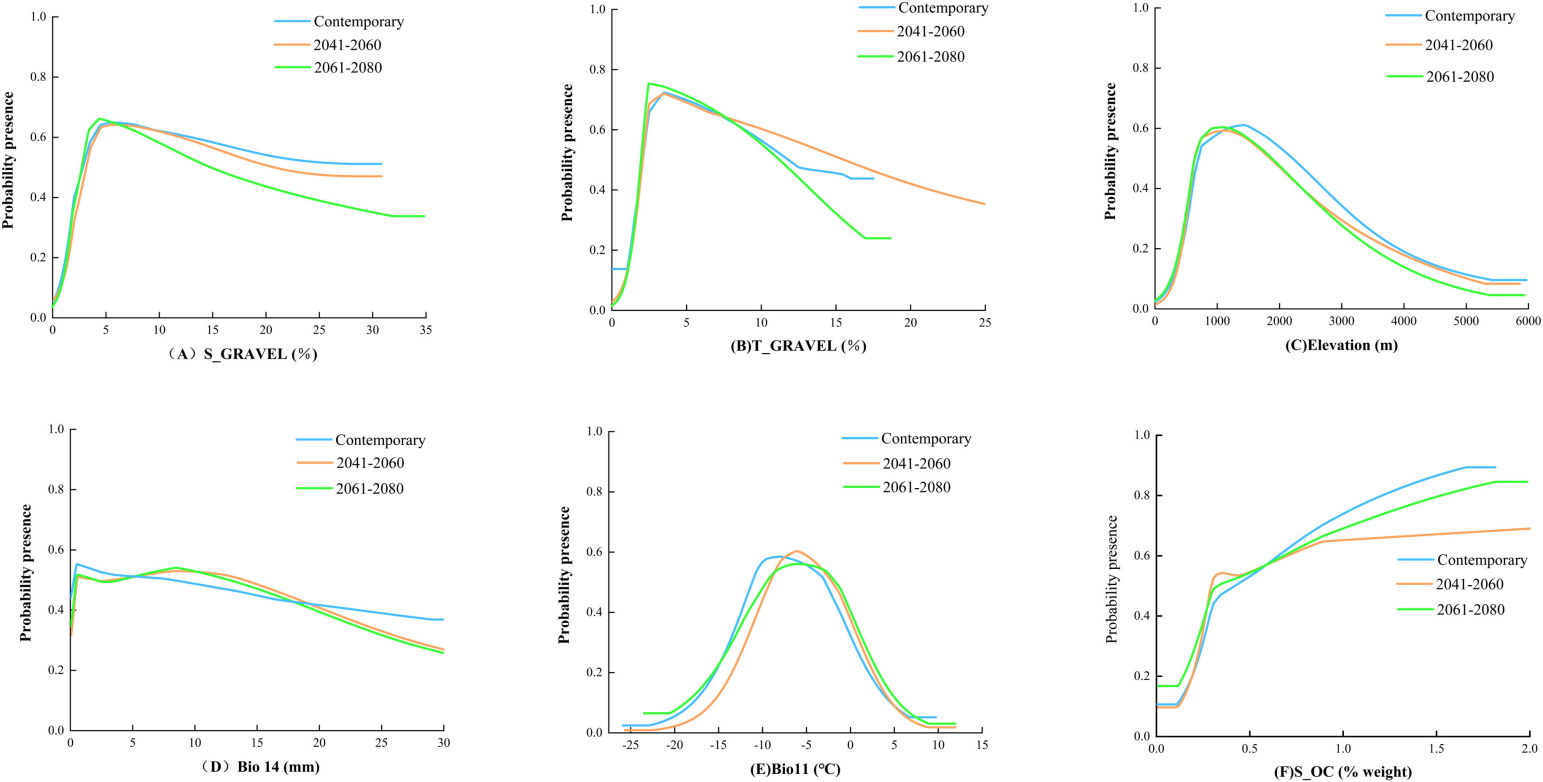
Response Curves of Probability of Presence to Main Environmental Factors.

The MaxEnt model’s environmental variable response curves (**Figure 3**; considering the parallel trends across the SSP1-2.6, SSP2-4.5, and SSP5-8.5 climate projections, only the SSP2-4.5 scenario is illustrated here for exemplification) elucidate the correlation of *B. tianschanica*’s occurrence likelihood with pivotal ecological variables. Generally, a plant’s presence probability exceeding 0.5 indicates suitable conditions for growth (Guo et al., 2019). Currently, optimal growth conditions for *B. tianschanica* include a lower soil gravel volume percentage between 2.8% and 29.8%, an upper soil gravel volume percentage between 2.1% and 11.8%, an elevation range of 695 to 2,211 m, an average temperature in the coldest quarter between −11.1°C and −2.8°C, and precipitation in the driest month ranging from 0.2 to 8.2 mm. Moreover, the presence probability of *B. tianschanica* is maximized when the lower soil organic carbon content exceeds 1.7%. Predictions for 2041–2060 suggest changes under suitable growth conditions: the lower soil gravel volume percentage will adjust to 3.1%–20.6%, the upper soil gravel volume percentage will range from 2.1% to 15.3%, the suitable elevation will decrease to 638–1,836 m, the average temperature in the coldest quarter will rise to −7.7°C to −1.1°C, and the precipitation in the driest month will increase to 0.5–13.9 mm, with the lower soil organic carbon content above 0.3%. Further projections for 2061–2080 indicate continued adjustments in growth conditions: the lower soil gravel volume percentage will be 2.8%–14.7%, the upper soil gravel volume percentage will be 1.9%–11.2%, the suitable elevation will be 650–1,877 m, the average temperature in the coldest quarter will range from −8.1°C to −0.7°C, the precipitation in the driest month will be 0.4–12.7 mm, and the lower soil organic carbon content will remain above 0.3%.

**Figure 4:**
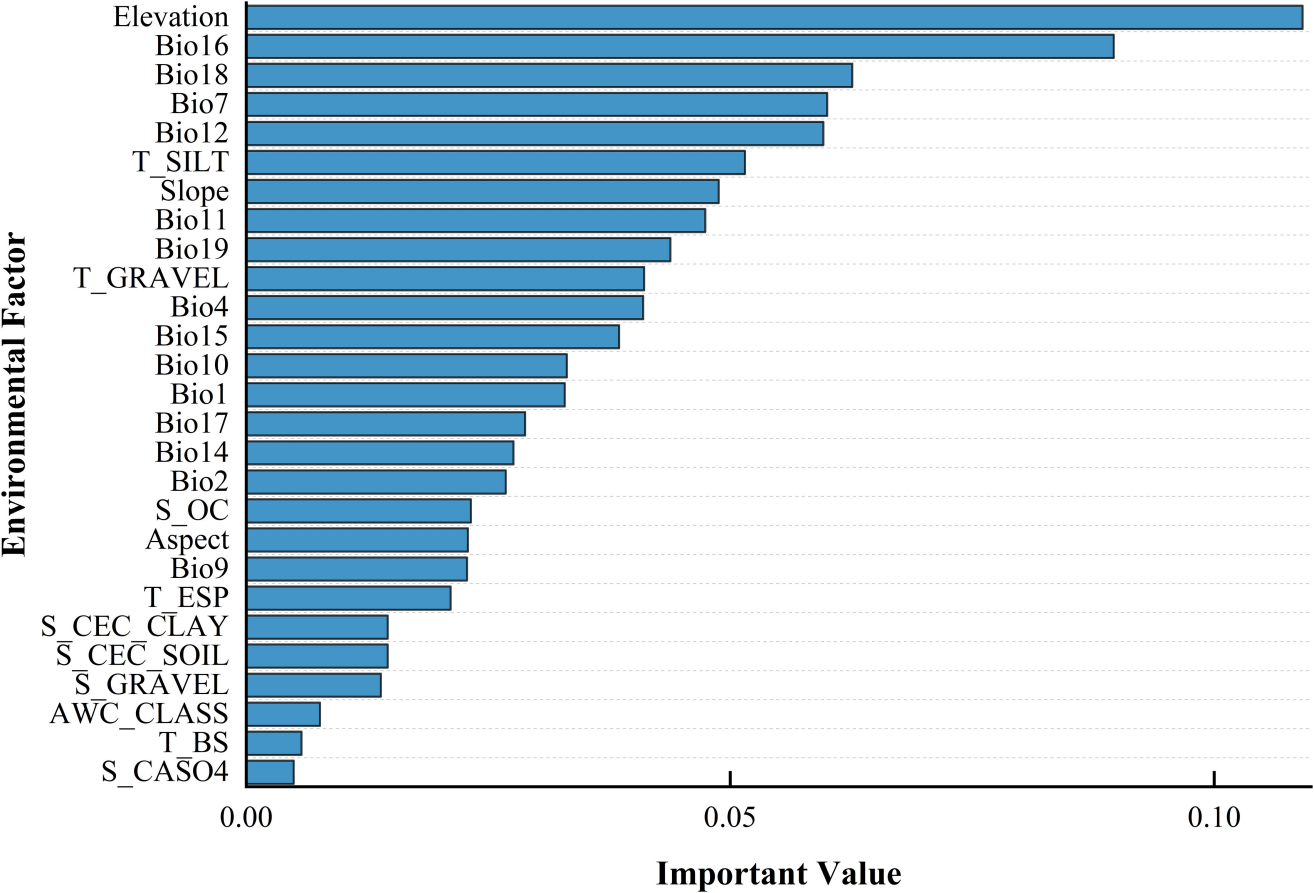
Ordering the Influence Intensity of Ecological Variables in Shaping the Geographic Spread of *Betula tianschanica*.

**Figure 5:**
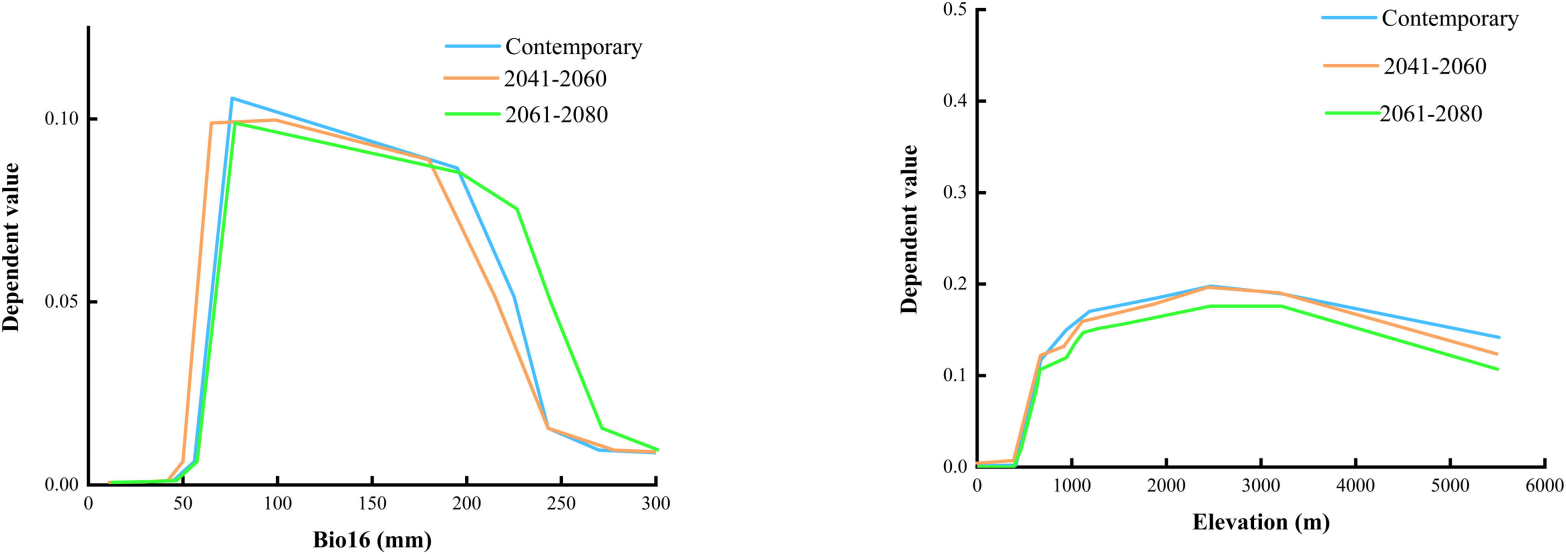
Environmental Factor Dependencies Output by Random Forest Model.

The outcomes of the RF model (**Figure 4**) suggest that terrain is the predominant influence on the occurrence of *B. tianschanica*, with climate playing a secondary role and soil factors exerting the weakest effect. In detail, altitude emerges as the most crucial terrain-related element, while the most pivotal climatic element is rainfall during the peak rainy period. Presently (**Figure 5**), *B. tianschanica* flourishes optimally at altitudes spanning 1,419 to 3,909 meters, accompanied by a wet season rainfall of 69 to 204 mm. Nevertheless, future projections for the period 2041–2060 anticipate a shift: the most favorable growth zones will migrate to higher altitudes, ranging from 1,878 to 3,639 m, with a reduction in wet season rainfall to 61–192 mm. Come 2061–2080, the region suitable for growth is anticipated to ascend further to altitudes between 2,386 and 3,266 m, alongside an uptick in wet season rainfall, varying from 70 to 225 mm.

### 3.2 Mapping suitable habitats under current climate conditions

**Figure 6:**
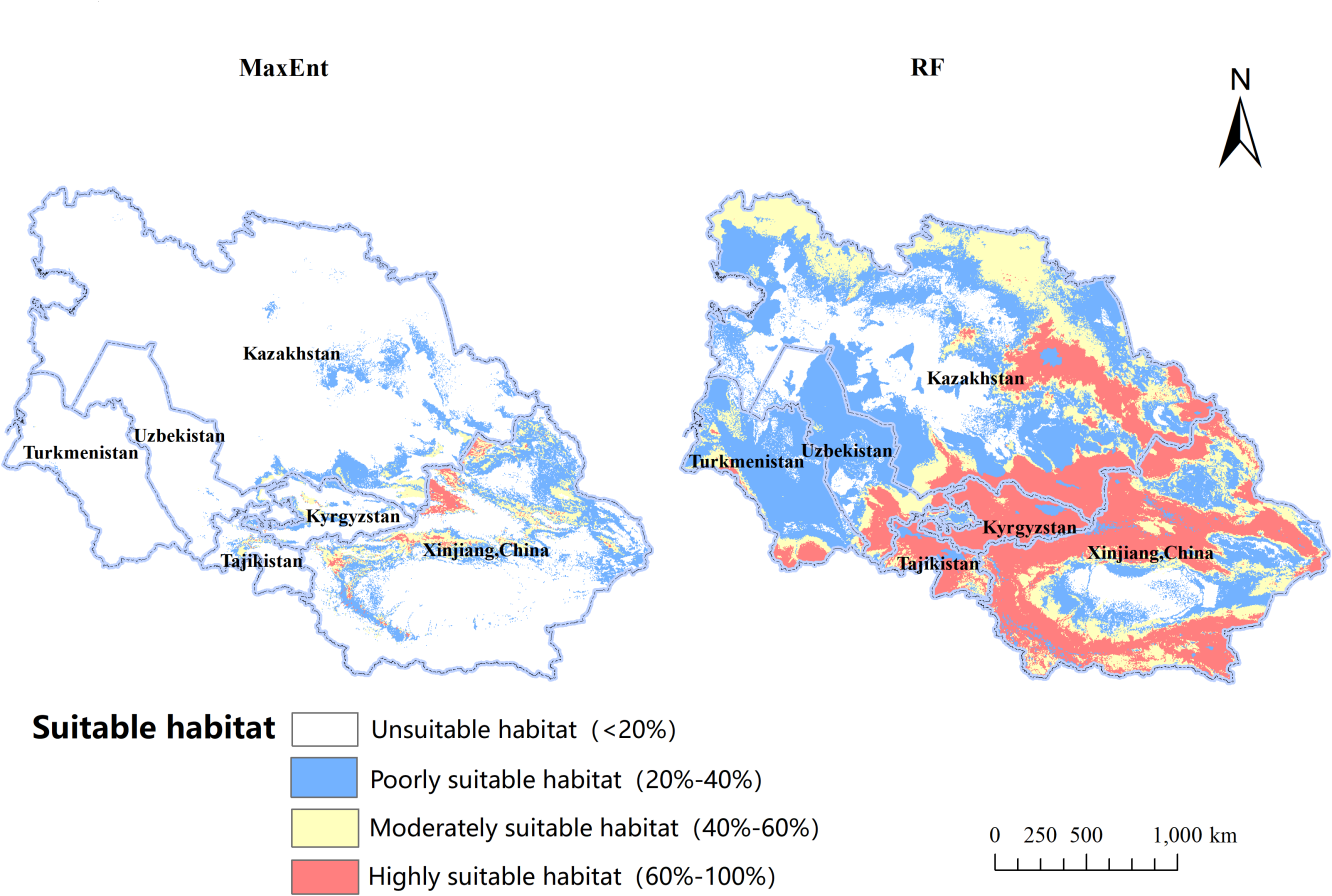
The Ecological Suitability Distribution of *Betula tianschanica* at Present.

Utilizing the MaxEnt algorithm, the projected occurrence map for *B. tianschanica* is depicted in **Figure 6**. Regions of prime habitat are predominantly found in the Tianshan Mountains, the Ili River Valley, around Lake Issyk-Kul, and among other locales, and are also scattered across the Turpan Basin, encompassing a zone of 5.81 × 10^4^ km^2^, which constitutes just 6.37% of the overall habitable zone. Zones of moderate habitat preference are largely concentrated in the Tianshan Mountains, along the Ili River, and within the Turpan Basin, covering an expanse of 22.03 × 10^4^ km^2^, representing 24.17% of the aggregate habitable zone. Areas with minimal habitat suitability exhibit an extensive spread, totaling 63.29 × 10^4^ km^2^, or 69.45% of the entire habitable zone. In addition to the Tianshan Mountains, these regions extend to the Irtysh River, Ulungur River, Bogda Mountains, Kazakh Uplands, Lake Balkhash, Amu River, and the midsection of the Syr River.

In contrast to the MaxEnt model, the cumulative suitable habitat for *B. tianschanica* as predicted by the RF model encompasses an area of 336.21 × 10^4^ km^2^, exceeding threefold the size estimated by the MaxEnt model and showing a more concentrated pattern. Within this, the prime habitat zone spans 89.55 × 10^4^ km^2^, predominantly located in the Tianshan Mountains, the Ili River Valley, Lake Balkhash, Lake Issyk-Kul, the Kazakh Uplands, along the Irtysh River, and the upper course of the Syr River; the zones of moderate habitat suitability are not only adjacent to the prime habitat zones but also extend into the lush regions at the southern periphery of the Karakum Desert, covering an area of 92.54 × 10^4^ km^2^; the least suitable habitats stretch eastward toward the environs of the Junggar Basin, northward toward the Turgay Depression and the Ural River Basin, and westward toward the southeastern shores of the Caspian Sea, encompassing an area of 154.12 × 10^4^ km^2^.

### 3.3 Prediction of suitable area under future climate scenario

**Figure 7:**
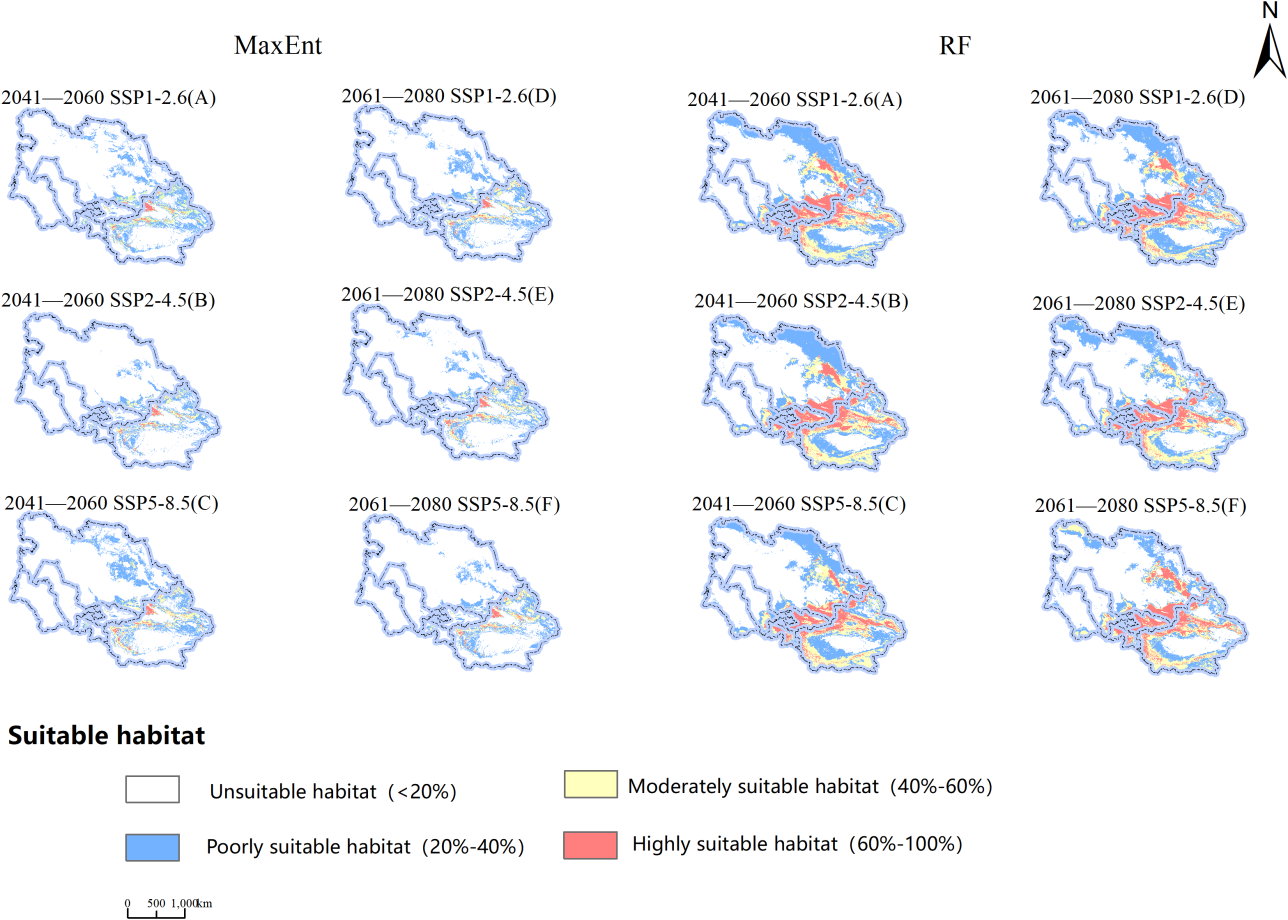
Projections of Potential Geographic Distribution of Betula tianschanica under Three Emission Scenarios for 2041—2060 **(A–C)** and 2061—2080 **(D–F)**.

Examining the information presented in **Figure 7** and **Table 4**, observations indicate variations in the viable cultivation zones for *B. tianschanica* across two timeframes (2041–2060 and 2061–2080) as predicted by the MaxEnt model under various climate projections. In the SSP1-2.6 and SSP5-8.5 scenarios, from the present era to 2041–2060, the aggregate suitable cultivation zone for *B. tianschanica* expanded by 21.58 × 10^4^ km^2^ and 38.87 × 10^4^ km^2^, respectively. The most substantial growth occurred under the SSP5-8.5 projection, expanding by 42.70%. However, under the SSP2-4.5 projection, the aggregate suitable zone in 2041–2060 measured 77.43 × 10^4^ km^2^, marking a 15.03% reduction from the current era. Progressing from the present to 2061–2080, the overall suitable zone for *B. tianschanica* experienced growth across varying climate projections, yet the rate of expansion waned as CO_2_ levels rose, with increases of 19.66%, 10.06%, and 4.98%. On the whole, the high- and moderate-suitability zones for *B. tianschanica* saw minimal change, predominantly situated in the Tianshan Mountains, along the Ili River, and in the Turpan Basin, whereas the low-suitability zones expanded toward the Kazakh Uplands.

**Table 4:** Extent of prospective habitat zones for *Betula tianschanica* across various temporal intervals/10^4^ km^2^.

Model	Period	Scenarios	Predicted area/104 km2				
			Poorly suitable habitat	Moderately suitable habitat	Highly suitable habitat	Total habitat	Change ratio/%
MaxEnt	Current	**━━**	63.29	22.03	5.81	91.13	**━━**
	2041–2060	SSP1-2.6	76.78	28.48	7.45	112.71	23.7%
		SSP2-4.5	52.01	19.78	5.64	77.43	−15.03%
		SSP5-8.5	89.29	33.37	7.34	130.00	42.7%
	2061–2080	SSP1-2.6	77.27	25.18	6.60	109.05	19.66%
		SSP2-4.5	64.03	28.32	7.95	100.30	10.06%
		SSP5-8.5	62.69	25.37	7.61	95.67	4.98%
RF	Current	**━━**	154.12	92.54	89.55	336.21	**━━**
	2041–2060	SSP1-2.6	159.18	96.34	76.27	331.79	−1.31%
		SSP2-4.5	155.92	100.47	81.44	337.84	0.48%
		SSP5-8.5	147.98	96.61	74.42	319.00	−5.11%
	2061–2080	SSP1-2.6	150.61	94.41	76.12	321.14	−4.48%
		SSP2-4.5	137.54	95.32	70.55	303.41	−9.76%
		SSP5-8.5	110.75	96.60	85.78	293.14	−12.81%

The outcomes of the RF model indicate that from the current era to the 2041–2060 timeframe, the overall viable region for *B. tianschanica* saw an expansion of 1.63 × 10^4^ km^2^ under the SSP2-4.5 projection, whereas it exhibited a downward trend across other timeframes or projections, particularly pronounced during the 2061–2080 period. This is primarily observed in the shrinkage of the least suitable zones in the Kazakh Uplands and the Turgay Depression region.

Upon analyzing the projected future habitat shifts as forecasted by both models, it is evident that aside from the contraction in the SSP2-4.5 projection (2041–2060) as per the MaxEnt model, there was a general trend of expansion in other projections and periods, with substantial rates of change. Conversely, the RF model displayed an inverse pattern. Beyond the growth in the SSP2-4.5 projection (2041–2060), there was a decline in habitat suitability across other projections and periods, with notable fluctuations in the rates of expansion and contraction.

## 4 Discussion

### 4.1 Model accuracy and prediction results

This research utilized the MaxEnt and RF algorithms, integrating climatic, edaphic, and morphological data from Central Asia, alongside the sampled occurrences of *B. tianschanica*, to investigate the impact of ecological variables on the prospective range of the species. The findings are considered credible. Field data for some species were collected through extensive surveys, with GPS technology used for precise location marking, enhancing the accuracy and dependability of the model beyond literature or database reliance. Both models demonstrated high predictive precision, with MaxEnt’s AUC exceeding 0.97 and RF’s AUC over 0.86, suggesting MaxEnt’s slightly superior performance. However, RF predicts a much broader potential distribution for *B. tianschanica*. This discrepancy may stem from differences in model assumptions and sample bias handling (MaxEnt alleviates sample bias through strategic selection and weight adjustment of background points, while RF employs bootstrap sampling methods to effectively reduce bias). MaxEnt, based on maximum entropy, may predict a more even distribution when data points are scarce or unevenly distributed, potentially underestimating actual species presence in certain areas, whereas RF behaves oppositely. The predicted primary distribution areas for *B. tianschanica* include the Tianshan Mountains, Ili River Basin, Issyk-Kul Lake, Turpan Basin, Irtysh River, Ulungur River, Bogda Mountain, Kazakh Hills, Balkhash Lake, Amu Darya, and the middle reaches of the Syr Darya River. The identified areas are consistent with previous studies (Ding et al., 2021), thereby enhancing the model’s reference value. Furthermore, areas outside the documented regions are identified as potential suitable habitats, where future climatic conditions are expected to support the growth of *B. tianschanica*.

### 4.2 Factors driving *B. tianschanica* distribution

The distribution of species is governed by various ecological elements, such as climatic conditions, in conjunction with soil and terrain characteristics (Wang and Xing, 2021; Wu and Wang, 2021). This study, focusing on *B. tianschanica*, employed the MaxEnt and RF models to analyze the relationship between its distribution pattern and these environmental factors. The MaxEnt model indicates that the key ecological elements influencing the occurrence of *B. tianschanica* are soil properties (such as the proportion of gravel in the lower and upper soil strata and the organic carbon content in the lower soil layer), climatic variables (including rainfall during precipitation of the driest month and mean temperature of the coldest quarter), and terrain attributes (specifically elevation). Presently, the favorable conditions for the growth of *B. tianschanica* are characterized by a lower soil gravel percentage of 2.8% to 29.8%, altitudes between 695 and 2,211 m, mean temperatures in the coldest quarter from −11.1°C to −2.8°C, and a range of 0.2 to 8.2 mm for precipitation in the driest month. The temperature and moisture needs of plants dictate their vertical distribution, which subsequently impacts individual development and the spatial pattern of tree species (Yu et al., 2016). The simulation forecasts alterations in the thermal and hydration requirements of *B. tianschanica* moving forward. The average temperature of the coldest quarter is projected to rise by roughly 2°C, prompting a shift to lower altitudes ranging from 650 to 1,877 m. Simultaneously, an enhanced necessity for hydration is anticipated, with the precipitation in the driest month anticipated to escalate to a range of 0.5 to 12.7 mm. Soil texture plays a pivotal role in determining water retention and aeration, both of which are essential for optimal root development and efficient nutrient absorption in plants (Benning and Moeller, 2021). Well-structured soil facilitates robust root growth, which in turn accelerates plant growth rates, improves overall health, and enhances adaptability to environmental conditions. To preserve soil moisture, it is projected that the volume percentage of gravel in the subsoil will decrease by approximately 15%. This adjustment aims to enhance the soil’s water-retention capabilities, thereby promoting healthier root development and superior overall plant performance. The random forest model’s projections reveal that the foremost ecological elements governing the spread of *B. tianschanica* encompass relief (elevation), meteorological conditions [precipitation of the wettest quarter, precipitation of the warmest quarter, temperature annual range (Bio5–Bio6), and annual precipitation], as well as soil attributes (proportion of silt). *B. tianschanica* exhibits optimal growth at altitudes between 1,419 and 3,909 m, where the precipitation of the driest quarter is from 69 to 204 mm during the current epoch. As CO_2_ concentrations escalate in the future, the vertical zone amenable to the proliferation of *B. tianschanica* will contract to a range of 2,386 to 3,266 m. Furthermore, the peak rainfall during the height of the rainy season is projected to increase, ranging from 70 to 225 mm. The two analytical models agree that *B. tianschanica* will exhibit heightened hydration needs in the coming years, whereas the forecasts for thermal requirements vary, resulting in divergent shifts in the optimal altitude spectrum for vegetation. These variances may stem from multiple causes. First, the temporal misalignment between bioclimatic and soil datasets can introduce significant biases in evaluating future plant growth conditions. Second, the study may not have fully accounted for all environmental determinants of plant growth, such as solar radiation and anthropogenic influences. Additionally, the limitations of data sources—namely, the coarse resolution (2.5-minute grid) and restricted temporal scope of the climate data—impede the accurate representation of microscale climatic variability and long-term climatic trends. Collectively, these issues may compromise the robustness and reliability of the predictive models.

## 5 Conclusion

This study utilizes the MaxEnt model and RF model to thoroughly evaluate the suitable habitats for *B. tianschanica*. The analysis identifies the percentage volume of gravel in the subsoil, elevation, mean temperature of the coldest quarter, precipitation during the driest month, and precipitation during the wettest quarter as critical determinants of its distribution. Significant dynamic shifts in suitable habitat areas are anticipated under future climate scenarios. Particularly under the SSP5-8.5 scenario for the period 2041–2060, the suitable habitat area is projected to expand by 42.7%, spreading eastward into Xinjiang, China, and toward central Kazakhstan.

In light of the findings yielded by this investigation, the following suggestions are put forth: a) designate protected areas within the Tianshan Mountains and limit human activities to reduce disturbances to the habitat of *B. tianschanica*. Special attention should be paid to protecting areas where the gravel volume percentage in the lower soil layer is between 2.8% and 29.8%, as elevations of 1,419–2,211 m represent key growth regions that require strengthened protective measures. b) Relocation conservation involves transplanting *B. tianschanica* to botanical gardens or seed banks for safekeeping from threats in their native environments. Additionally, attempts at artificial propagation under suitable growth conditions should be made to increase population numbers. c) Establish long-term ecological and behavioral research stations within the protected area to monitor the population dynamics of *B. tianschanica*. This monitoring will provide a scientific basis for conservation strategies. Special attention should be given to the growth conditions of *B. tianschanica* when the mean temperature of the coldest quarter ranges from −11.1°C to −2.8°C, the precipitation of the driest month is between 0.2 and 8.2 mm, and the precipitation of the wettest quarter is between 69 and 204 mm. d) Strengthen international cooperation by jointly developing conservation plans and establishing transnational protected areas along the Belt and Road Initiative to collectively protect the ecological environment of *B. tianschanica*.

## Data Availability

The original contributions presented in the study are included in the article/supplementary material. Further inquiries can be directed to the corresponding author.
